# Mining Symptom-Herb Patterns from Patient Records Using Tripartite Graph

**DOI:** 10.1155/2015/435085

**Published:** 2015-06-08

**Authors:** Jinpeng Chen, Josiah Poon, Simon K. Poon, Ling Xu, Daniel M. Y. Sze

**Affiliations:** ^1^School of Computer Science and Engineering, BeiHang University, Beijing, China; ^2^School of Information and Technologies, University of Sydney, Sydney, NSW, Australia; ^3^Shanghai University of Traditional Chinese Medicine, Shanghai, China; ^4^RMIT University, Melbourne, VIC, Australia

## Abstract

Unlike the western medical approach where a drug is prescribed against specific symptoms of patients, traditional Chinese medicine (TCM) treatment has a unique step, which is called syndrome differentiation (SD). It is argued that SD is considered as patient classification because prior to the selection of the most appropriate formula from a set of relevant formulae for personalization, a practitioner has to label a patient belonging to a particular class (syndrome) first. Hence, to detect the patterns between herbs and symptoms via syndrome is a challenging problem; finding these patterns can help prepare a prescription that contributes to the efficacy of a treatment. In order to highlight this unique triangular relationship of symptom, syndrome, and herb, we propose a novel three-step mining approach. It first starts with the construction of a heterogeneous tripartite information network, which carries richer information. The second step is to systematically extract path-based topological features from this tripartite network. Finally, an unsupervised method is used to learn the best parameters associated with different features in deciding the symptom-herb relationships. Experiments have been carried out on four real-world patient records (Insomnia, Diabetes, Infertility, and Tourette syndrome) with comprehensive measurements. Interesting and insightful experimental results are noted and discussed.

## 1. Introduction

Traditional Chinese medicine (TCM) has a long history and has been accepted as one of the main medical approaches in China [1]. Many of the herbal medicines used in today's clinical practice and some of the traditional Chinese medicine preparation has been used in human patients for thousands of years, which has been successfully applied to the treatment of many diseases, such as insomnia, diabetes, infertility, and Tourette syndrome. Unlike the western medical approach where a drug is prescribed against specific symptoms of patients, TCM treatment has a unique step, which is called syndrome differentiation (SD). It is argued that SD is, in fact, patient classification because, prior to the personalization of the most appropriate formula, a practitioner has to label a patient belonging to a particular class (syndrome) for a set of relevant formulae. Hence, to detect the patterns between herbs and symptoms via syndrome is a challenging problem; finding these patterns can help prepare a prescription that contributes to the efficacy of a treatment.

In recent years, interest in TCM has increased globally and the application of data mining to TCM [2–4] is also getting more attention. However, most of the previous research was related to the extraction of core herbs or to mine herb-herb relationships [1, 5, 6] from a network of herbs. We term this kind of network as a homogeneous information network, that is, network consisting of only one type of objects (herb in this example). When a network contains different types of objects (such as herbs, symptoms, and syndromes), we refer to them as heterogeneous information networks. Since heterogeneous information networks are not well studied, this has become the motivation of our work.

In general, a homogeneous information network can be derived from a heterogeneous information network, for example, an herb-herb network can be derived from a symptom-syndrome-herb network by a projection on herbs only. A heterogeneous information network is different from a homogeneous information network because it carries richer information than its corresponding projected homogeneous information networks. Therefore, it aimed to discover herb-symptom patterns, via syndromes, from a heterogeneous information network, which contains different types of attribute values associated with objects. To the best of our knowledge, this is the first attempt towards mining herb-symptom patterns in TCM utilizing heterogeneous information networks.

In this research, we construct the heterogeneous information network leveraging the tripartite graph. Our heterogeneous information network contains multiple types of objects, such as herb, symptom, syndrome, and multiple types of links defining different relations among these objects, such as links existing between herbs and syndromes, between syndromes and symptoms, and between symptoms and herbs. Thus, the number of different types of objects there are in the network can be found out, as well as the identification of the possible links existing among objects. Furthermore, we can detect the patterns between herbs and symptoms.

The major contributions of this paper are summarized.

1. We construct the TCM heterogeneous information network utilizing the tripartite graph.
2. We study the problem of the symptom-herb relationship prediction in TCM heterogeneous information network.
3. We propose a novel three-step prediction approach based on the TCM heterogeneous information network to discover symptom-herb patterns.
4. Experiments on real TCM patient records indicate that our proposed method can mine symptom-herb relationships with high accuracy.
5. Treatments are proven to be more effective than a direct symptom-herb relationship; that is, classifying patients into different syndromes is a crucial step in TCM treatment.

The remaining of the paper is organized as follows. We first introduce the background and preliminaries on TCM heterogeneous information networks and denote the task of symptom-herb pattern prediction in Section 2. In Section 3, we obtain some interesting observations based on TCM heterogeneous information network. We next present a novel three-step mining approach to discover the symptom-herb patterns in Section 4. We report our experiments and results in Section 5, discuss related work in Section 6, and conclude the study in Section 7.

## 2. Preliminaries and Problem Definition

### 2.1. Notations Definitions

In this work, we need to consider three types of entities: a set of herbs *H* = {*h*_1_, *h*_2_,…, *h*_*n*_}, a set of syndromes *D* = {*d*_1_, *d*_2_,…, *d*_*m*_}, and a set of symptoms *P* = {*p*_1_, *p*_2_,…, *p*_*q*_}. We assume that there are *n* herbs, *m* syndromes, and *q* symptoms. Here, symptoms refer to something that can be observed and measured, such as fever, nausea, coughing, and weight loss. Syndrome is a special phenomenon in TCM. A TCM doctor will base upon the patient's symptoms and classify them into one or two syndromes. After that, formulas will be prescribed according to the syndrome.

### 2.2. Heterogeneous Information Network

We first introduce the definitions of heterogeneous information network [7, 8], tripartite graph [9], and tritype information network, so as to study the characteristic of TCM and discuss how to find or predict symptom-herb patterns in TCM information network.

**Definition 1 (heterogeneous information network).**

A heterogeneous information network is denoted as a directed graph *G* = (*V*, *E*, *W*) with an entity type mapping function *ϕ* : *V* → *𝒜* and a link type mapping function *ψ* : *E* → *ℛ*, where each entity *v*⊆*V* belongs to one particular entity type *ϕ*(*v*)⊆*𝒜*, each link *e*⊆*E* belongs to a particular relation type *ψ*(*e*)⊆*ℛ*, and *W* : *E* → *R* ^+^ is a weight mapping from an edge *e*⊆*E* to a real number *w*⊆*R* ^+^. Notice that, when the types of entities |*𝒜*| > 1 and also the types of relations |*ℛ*| > 1, the network is called heterogeneous information network.

**Definition 2 (tripartite graph).**

A graph TG = 〈{*V*_1_ ∪ *V*_2_ ∪ *V*_3_}, *E*〉 can be called as tripartite, if a set of graph nodes decomposed into three disjoint sets such that no two graph nodes within the same set are adjacent; that is, *V*_1_∩*V*_2_∩*V*_3_ = *∅*.

**Definition 3 (tritype information network).**

Given three types of objects sets *X*, *Y*, and *Z*, where *X* = {*x*_1_, *x*_2_,…, *x*_*m*_}, *Y* = {*y*_1_, *y*_2_,…, *y*_*n*_}, and *Z* = {*z*_1_, *z*_2_,…, *z*_*q*_}, graph *G* = 〈*V*, *E*〉 is called a tritype information network on types *X*, *Y*, and *Z*, if *V*(*G*) = *X* ∪ *Y* ∪ *Z* and *E*(*G*) = {〈*o*_*i*_, *o*_*j*_〉}, where *o*_*i*_, *o*_*j*_ ∈ *X* ∪ *Y* ∪ *Z*.

Let *W*_(*m*+*n*)*∗*(*m*+*n*)_ = {〈*w*_*o*_*i*_*o*_*j*__〉} (or *W*_(*n*+*q*)*∗*(*n*+*q*)_ = {〈*w*_*o*_*i*_*o*_*j*__〉} or *W*_(*m*+*q*)*∗*(*m*+*q*)_ = {〈*w*_*o*_*i*_*o*_*j*__〉}) be the adjacency matrix of links, where 〈*w*_*o*_*i*_*o*_*j*__〉 equals the weight of link 〈*o*_*i*_, *o*_*j*_〉, which is the observation number of the link, and we thus use *G* = 〈{*X* ∪ *Y* ∪ *Z*}, *W*〉 to define this tritype information network with weight. In the following, we use *X*, *Y*, and *Z* denoting the object set and their type name. For convenience, we decompose the link matrix into four blocks: *W*_*XX*_, *W*_*XY*_, *W*_*YX*_, and *W*_*YY*_ (or *W*_*YY*_, *W*_*YZ*_, *W*_*ZY*_, and *W*_*ZZ*_ or *W*_*XX*_, *W*_*XZ*_, *W*_*ZX*_, and *W*_*ZZ*_), each denoting a subnetwork of objects between types of the subscripts. *W* can be denoted as

(1) $$\begin{array}{c} W=\left(\begin{array}{cc} W_{XX} & W_{XX}\\ W_{YX} & W_{YY} \end{array}\right)\;or\;W=\left(\begin{array}{cc} W_{YY} & W_{YZ}\\ W_{ZY} & W_{ZZ} \end{array}\right)\\ or\;\;W=\left(\begin{array}{cc} W_{XX} & W_{XZ}\\ W_{ZX} & W_{ZZ} \end{array}\right). \end{array}$$

**Figure 1:**
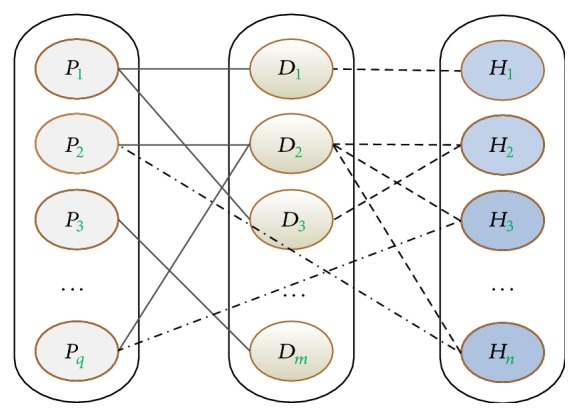
Tripartite graph structure of TCM. Here, instances of different objects are represented by different colour nodes and links among different objects are represented by different line styles. *q*, *m*, and *n* represent the number of symptom, the number of syndrome, and the number of herb, respectively.

This tritype information network, one of the heterogeneous information networks, denotes the rules of how entities exist and how links should be created. And, through analyzing this tritype information network, we can know how many types of objects there are in the network and where the possible links exist. In the following, we give an example of tritype information network, which is showed in Figure 1. Here, as an abbreviation, we utilize the special letters to define these entity types, namely, *H* representing herbs, *P* representing symptoms, and *D* representing syndromes. Notations and similarity relations used in definitions as well as the rest part of the paper can be found in Notation section.

### 2.3. Target Relationship Prediction

Based on the previous definitions, our goal of this work can be summarized as follows: given a tritype network *G* = 〈{*H* ∪ *D* ∪ *P*}, *W*〉, the target type *P*, and a set of herbs {*H*_*j*_}, our goal is to find or predict the most reasonable herbs for each symptom *P*_*i*_, that is, how to predict the target relationship *E*(*G*) = {〈*P*_*i*_, *H*_*j*_〉}, where *P*_*i*_, *H*_*j*_ ∈ *P* ∪ *H*.

Different from symptom-syndrome patterns and syndrome-herb patterns, which are directed relationships (because patients' syndromes are derived from a set of patients symptoms and herbs are configured by doctors according to the patients' syndromes, symptom-syndrome patterns and syndrome-herb patterns are directed relationships.), symptom-herb patterns are undirected relationships. Intuitively, the herb-symptom relationship detection is an implicit relationship mining, which is more difficult to detect than an explicit relationship mining. However, if new herb-symptom relationships can be discovered, they are beneficial for doctors configuring the prescriptions.

### 2.4. Dataset

In this work, our experiments were performed on four real TCM datasets: Insomnia, Infertility, Diabetes, Tourette. These four datasets were provided by Guang'anmen Hospital, China Academy of Chinese Medical Sciences. These four datasets include the symptoms, the syndromes, and prescription information of outpatients. Here, edges are formed among objects belonging to the same prescription. Properties of these four datasets are shown in Table 1.

## 3. Observation

In this section, we conduct following observations based on the four TCM datasets in order to get a better understanding on the symptom-syndrome-herb patterns and structural properties of TCM tripartite network.

### 3.1. Entity Distribution

**Figure 2:**
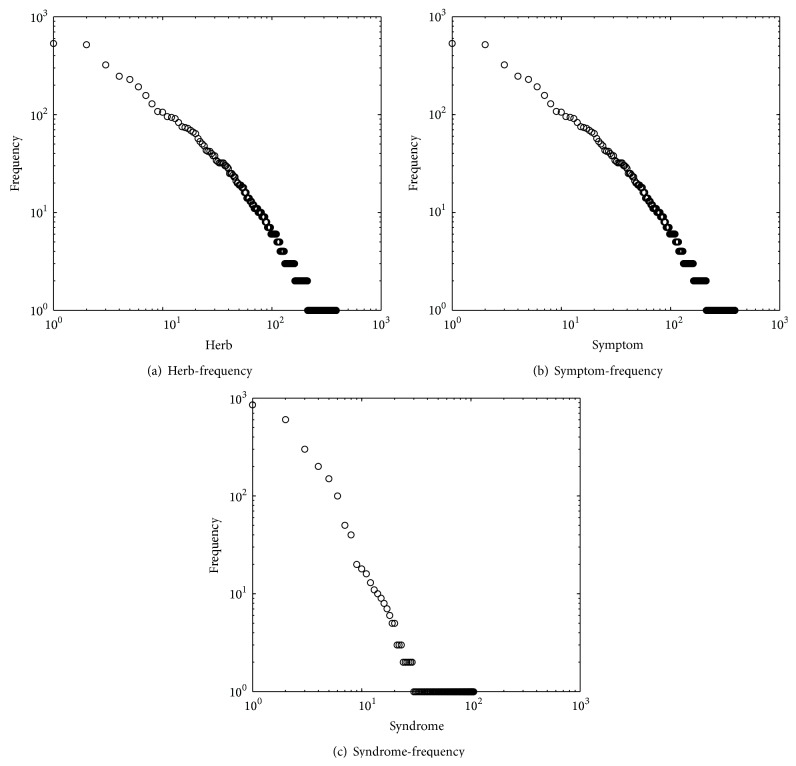
Distribution of the entity frequency in Infertility Dataset. Here, in (a), the *x*-axis represents the 251 unique herbs, ordered by descending herb frequency. The *y*-axis refers to the herb frequency. In (b), the *x*-axis represents the 389 unique symptoms, ordered by descending symptom frequency. The *y*-axis refers to the symptom frequency. In (c), the *x*-axis represents the 106 unique syndromes, ordered by descending syndrome frequency. The *y*-axis refers to the syndrome frequency.

We first study the distribution of each entity frequency.  Figure 2 plots the distribution in a log-log scale based on the Infertility dataset. In Figure 2(a), the *x*-axis represents the 251 unique herbs, ordered by descending herb frequency. The *y*-axis refers to the herb frequency. As reported by other authors [5, 10], we find the herb frequency to follow a power law distribution with few herbs being responsible for a high number of prescriptions. Here, the probability of a kind of herb having herb frequency *x* is proportional to *x* ^−0.843^. It indicates that most herbs are rarely used, while only a small number of the herbs are frequently used. In other words, the head of the power law contains herbs that would be used more frequently and the very tail of the power law contains the infrequent herbs. The most frequent herbs were used more than 530 times by different prescriptions altogether. Similarly, same distributions can be found in Figures 2(b) and 2(c).

In addition to the infertility dataset, we carried on similar statistical analysis with other three datasets, and the same pattern is observed in the vast majority of cases.

### 3.2. Link Distribution

So far, there is some existing work that explicitly addresses herb-herb patterns [5, 6]. They indicated that there are common herb pairs frequently used in the regular TCM herb prescriptions. However, few works focus on studying symptom-herb, symptom-syndrome, and syndrome-herb patterns. In this work, we extract these patterns and analyze what distribution they obey.

**Figure 3:**
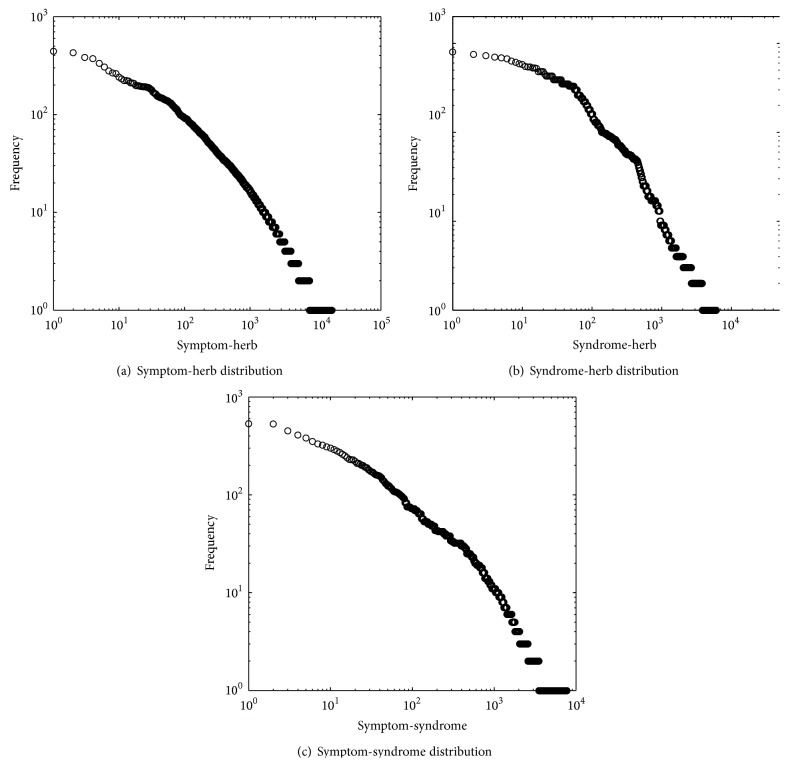
Distribution of the link frequency in Infertility Dataset. Here, in (a), the *x*-axis represents the 17,910 symptom-herb patterns, ordered by descending symptom-herb frequency. The *y*-axis refers to the symptom-herb frequency. In (b), the *x*-axis represents the 6,085 syndrome-herb patterns, ordered by descending syndrome-herb frequency. The *y*-axis refers to the syndrome-herb frequency. In (c), the *x*-axis represents the 7,897 symptom-syndrome patterns, ordered by descending symptom-syndrome frequency. The *y*-axis refers to the symptom-syndrome frequency.

Figure 3 shows that the distribution of these patterns (symptom-herb, symptom-syndrome, and syndrome-herb patterns) also follows a power law distribution. In Figure 3(a), the *x*-axis represents the 17,910 symptom-herb patterns, ordered by their cooccurrence frequency (descending). The *y*-axis refers to the symptom-herb frequency. Furthermore, we find that 80% of all symptom-herb patterns appear only 1–3 times in the infertility dataset. Here, the probability of a kind of symptom-herb pattern having symptom-herb pattern frequency *x* is proportional to *x* ^−0.945^. This indicates that there are common herb-symptom pairs frequently used in the regular TCM herb prescriptions. If we can predict these common herb-symptom pairs, it is very useful for a doctor configuring a formulae. Again, the same law distributions can be found in Figures 3(b) and 3(c).

### 3.3. Relationship Distribution

**Figure 4:**
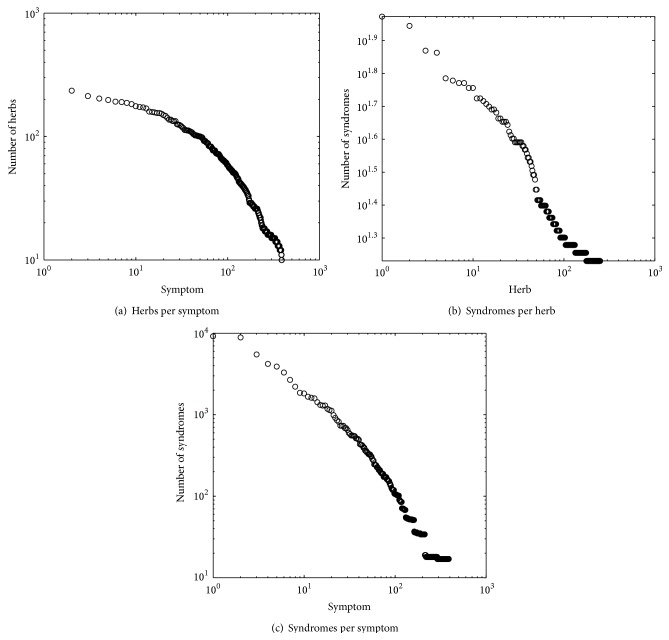
Distribution of relationship of objects in Infertility Dataset. Here, in (a), the *x*-axis represents the 389 unique symptoms, ordered by the descending number of herbs per symptom. The *y*-axis refers to the number of herbs per symptom. In (b), the *x*-axis represents 251 unique herbs, ordered by descending number of syndromes per herb. The *y*-axis refers to the number of syndromes per herb. In (c), the *x*-axis represents the 389 unique symptoms, ordered by the descending number of syndromes per symptom. The *y*-axis refers to the number of syndromes per symptom.

Furthermore, we study the relationship among symptom, syndrome, and herb. Here, the relationship also exists among symptom, syndrome, and herb. It is a one-to-many relationship, that is, the number of herbs each symptom is associated with, the number of syndromes each herb is associated with, and so forth.  Figure 4 shows that the distribution of the number of herbs per symptom (syndromes per herb or syndromes per symptom) also follows a power law distribution. In Figure 4(a), the *x*-axis represents the 389 unique symptoms, ordered by the number of herbs per symptom (descending). The *y*-axis refers to the number of herbs per symptom. The probability of having *x* herbs per symptom is proportional to *x* ^−0.51^. We can find each symptom to be labeled with 46.4 herbs on average. Also, it can be found for the occurrence frequencies of herbs per symptom where 23.2% of all herbs link to the Top 1% of symptoms. Similarly, the same law distributions can be found in Figures 4(b) and 4(c).

## 4. Prediction Method Based on Tripartite Graph

In this section, we will introduce a novel three-step prediction approach based on the tripartite graph (Tri-TSPA). First, we extract two types of paths, which carry different semantic meanings. In terms of these two paths, we draw three matrices, which represent different cooccurrence relationship. And then, we propose an unsupervised prediction method in order to discover symptom-herb patterns.

### 4.1. Extracting Paths

In a tripartite network, two entities can be connected by different paths, which carry different semantic meanings. In this work, we choose two kinds of paths in order to find the reasonable symptom-herb patterns. These two kinds of paths are taken as follows:

(2) $$\begin{array}{c} PH\_Path\text{:}\;Symptom⟶Herb\\ PDH\_Path\text{:}\;Symptom⟶Syndrome⟶Herb. \end{array}$$

Path *PH*_Path extracts the direct target relationship; it looks like the way western medicine often adopts. In western medicine, medical doctors and other healthcare professionals (such as nurses, pharmacists, and therapists) treat diseases using drugs, radiation, or surgery according to symptoms [11]. Path *PDH*_Path extracts the indirect target relationship, it is a common way TCM often adopts. In TCM, doctors first choose a series of syndromes in terms of patients' symptoms, and, then, configure herbs on the basis of syndromes.

### 4.2. Constructing Matrix

After extracting paths from the tripartite graph, we can further construct matrices describing the relationship among different entities, such as symptom-herb, symptom-syndrome, and syndrome-herb. In this work, we build the three matrices, namely, symptom-herb matrix based on the path *PH*_Path, symptom-syndrome matrix, and syndrome-herb matrix based on the path *PDH*_Path.

In addition, we also build matrices depicting the relationship among same entities, such as herb-herb, symptom-symptom, and syndrome-syndrome, in order to promote the similarity measure and find some useful symptom-herb patterns. These three matrices can be extracted based on the homogeneous information networks (here, if two herbs (or symptoms, syndromes) belong to the same prescription and they produce the positive effect when used together, we can connect these two herbs. According to this rule, the homogeneous information networks can be constructed), including herb, symptom, and syndrome homogeneous information networks.

In order to build aforementioned matrices, we define and implement multiple measurement strategies in this work. These strategies can be introduced as follows.

 (i) * Frequency* (*F*). Frequency is a basic strategy, which is an observation number of cooccurrence of two entities (*A*_*x*_ and *A*_*y*_), such as symptom-herb, symptom-syndrome, and syndrome-herb. It can be defined as *F*(*A*_*x*_, *A*_*y*_):
  (3) $$\begin{array}{c} F\left(A_{x},A_{y}\right)=\left|\left\{\langle A_{x},A_{y}\rangle :A_{x},A_{y}\in P\cup D\cup H\right\}\right|. \end{array}$$
 (ii) * Jaccard Coefficient* (JC). According to the Jaccard coefficient [12], we can normalise the cooccurrence of two entities *A*_*x*_ and *A*_*y*_ by calculating
  (4) $$\begin{array}{c} JC\left(A_{x},A_{y}\right)=\frac{\left|A_{x}\cap A_{y}\right|}{\left|A_{x}\cup A_{y}\right|}. \end{array}$$
 The coefficient takes the number of intersections between the two entities, divided by the union of the two entities. The Jaccard coefficient is known to be useful to measure the relevance between two objects or sets. In general, we can use symmetric measures, like Jaccard, to induce whether two entities have a related meaning.
 (iii) * Asymmetric Measure* (AM). The cooccurrence of two entities *A*_*x*_ and *A*_*y*_ can be normalised leveraging the frequency of one of the entities [13–15], for instance, using equation
  (5) $$\begin{array}{c} AM\left(A_{x}\;|\;A_{y}\right)=\frac{\left|A_{x}\cap A_{y}\right|}{\left|A_{y}\right|}. \end{array}$$
 AM captures how often the entity *A*_*y*_ cooccurs with entity *A*_*x*_ normalised by the total frequency of entity *A*_*y*_. We can interpret this as the probability of a patient being diagnosed with entity *A*_*x*_ given entity *A*_*y*_ occuring.
 (iv) TfIdf. It is often used as a weighting factor in information retrieval and text mining [16]. In this work, we denote Tf(*A*_*x*_, *A*_*y*_) = *F*(*A*_*x*_, *A*_*y*_), which is the frequency of two entities (*A*_*x*_ and *A*_*y*_) cooccurrence and define Idf(*A*_*x*_, *A*_*y*_) = log⁡(*N*/*F*(*A*_*x*_, *A*_*y*_)), which measures the importance of *A*_*x*_-*A*_*y*_ patterns for the entity *A*_*x*_ (or *A*_*y*_). Thus, TfIdf(*A*_*x*_, *A*_*y*_) can be denoted as follows:
  (6) $$\begin{array}{c} TfIdf\left(A_{x},A_{y}\right)=F\left(A_{x},A_{y}\right)\log\frac{N}{F\left(A_{x},A_{y}\right)}, \end{array}$$
 where *N* is the frequency of *A*_*x*_  (or *A*_*y*_).

### 4.3. Symptom-Herb Patterns Prediction Method

In this subsection, we first show two similarity measures. And then, we introduce a relevance function. Finally, we proposed an unsupervised prediction method.

#### 4.3.1. Similarity Measures

A similarity measure is a real-valued function that quantifies the similarity between two objects. In this work, taking the symptom as an example, if two symptoms are similar, they are likely to have similar frequency of symptom-herb patterns. Given symptom *p*_1_, *p*_2_, and herb *h*_1_, if *p*_1_ is similar to *p*_2_, and there exists the *p*_1_-*h*_1_ pattern, we can infer that there exists the pattern *p*_2_-*h*_1_.

As mentioned previously, we have extracted two kinds of paths and built three matrices. Also, we have built other three homogeneous matrices. Based on them, we proposed two strategies measuring the similarity of entities of the same type.

 (i) *PH*_Path based similarity: On basis of the symptom-herb matrix and symptom-symptom matrix, we use cosine similarity sim*PH* and sim*PP* to compute symptoms similarity, respectively. By combining sim*PH* and sim*PP*, we can get *PH*_Path based similarity. It can be denoted as
  (7) $$\begin{array}{c} simPH\_Path\left(p_{x},p_{y}\right)=\lambda_{0}simPH+\lambda_{1}simPP, \end{array}$$
 where *λ*_0_, *λ*_1_ > 0 and *λ*_0_ + *λ*_1_ = 1. sim*PH* reflects the frequency similarity of symptom-herb patterns. In other words, if two symptoms are similar, they are likely to have similar frequency of symptom-herb patterns. sim*PP* reflects the frequency similarity of symptom-symptom patterns. In other words, if two symptoms belong to the same prescription, they are likely to be similar.
 (ii) *PDH*_Path based on similarity: In terms of the symptom-syndrome matrix, syndrome-herb matrix, and syndrome-syndrome matrix, we can obtain two syncretic syndrome similarities, sim*PDH*_1_(*d*_*x*_, *d*_*y*_) and sim*PDH*_2_(*d*_*x*_, *d*_*y*_). Furthermore, through combining these two syncretic syndrome similarities, *PDH*_Path based on similarity can be formalized as
  (8) $$\begin{array}{c} simPDH\_Path\left(d_{x},d_{y}\right)=\alpha simPDH_{1}+\beta simPDH_{2}, \end{array}$$
 where the definition of sim*PDH*_1_ and sim*PDH*_2_ is simlar to sim*PH*_Path, but their only difference is that sim*PDH*_1_ and sim*PDH*_2_ are based on the symptom-syndrome matrix, syndrome-herb matrix, and syndrome-syndrome matrix. Here, sim*PDH*_1_(*d*_*x*_, *d*_*y*_) = *α*_0_sim*PD*(*d*_*x*_, *d*_*y*_) + *α*_1_sim*DD*(*d*_*x*_, *d*_*y*_) and sim*PDH*_2_(*d*_*x*_, *d*_*y*_) = *β*_0_sim*DH*(*d*_*x*_, *d*_*y*_) + *β*_1_sim*DD*(*d*_*x*_, *d*_*y*_). Note that, *α*, *α*_0_, *α*_1_, *β*, *β*_0_, *β*_1_ > 0 and *α* + *β* = 1 and *α*_0_ + *α*_1_ = 1, *β*_0_ + *β*_1_ = 1.

#### 4.3.2. Relevance Function

In our datasets, the outcomes of all the prescriptions are classified into two categories: good and bad. When a treatment was effective, which means that if the patient recovered completely or partly from diseases in the next encounter, then the prescription of the current encounter would be categorized as “good”; otherwise, the prescription would be categorized as “bad.” In other words, when the outcome of a prescription is good, the patterns in this prescription, such as symptom-herb, symptom-syndrome, herb-herb, and others, make the positive role; otherwise, the patterns make a negative role.

In this work, relevance function is used to filter out the patterns with bad outcome. Here, the relevance function is parameterized with “relevance threshold” *θ* ∈ [0,1] to provide a range of tolerance to bad outcomes. In particular, given a relevance function *R*(〈*A*_*x*_, *A*_*y*_〉∣*θ*), the relevance threshold *θ* is used for creating the parameterized version of this relevance function, *R*(〈*A*_*x*_, *A*_*y*_〉∣*θ*), that is formalized as

(9) $$\begin{array}{c} R=\left\{\begin{array}{cc} 1 & if\;\;A_{x}\cap A_{y}\neq \emptyset ,\;\;ratio\in \left(\left.\theta ,1\right]\right.\\ 0 & else, \end{array}\right. \end{array}$$

where *θ* changes over different datasets. *A*_*x*_, *A*_*y*_ ∈ *X* ∪ *Y* ∪ *Z* and ratio = Good_Outcome(Pattern)/(Good_Outcome(Pattern) + Bad_Outcome(Pattern)). Here, Good_Outcome(Pattern) refers to the total number of this pattern working effectively, and Bad_Outcome(Pattern) is the total number of this pattern having no effect on patients. In the next section, patterns of symptom-herb that are predicted above relevance threshold *θ* (i.e., *R*(〈*A*_*x*_, *A*_*y*_〉∣*θ*) = 1) are sorted according to predicted rating, while patterns of symptom-herb that are below *θ* (i.e., *R*(〈*A*_*x*_, *A*_*y*_〉∣*θ*) = 0) are ignored.

#### 4.3.3. Proposed Method

Up to now, we have given a systematic way to extract and build the topological features in the tripartite networks. In this subsection, we will introduce our prediction algorithm (Tri-TSPA). Our prediction method is as follows: first, we discover *K* nearest entities according to the similarity measures, sim*PH*_Path(*A*_*x*_, *A*_*y*_) or sim*PDH*_Path(*A*_*x*_, *A*_*y*_); then, we predict rating for each potential entity pair; subsequently, we get Top-*n* predicted patterns by ranking prediction rating; lastly, we get Top-*N* list by filtering the patterns of bad outcome using relevance function. The pseudocode of Tri-TSPA is shown in Algorithm 1.

In Algorithm 1, we only show the *F* measurement strategy to calculate the rating. Actually, we can replace *F*(·, ·) with JC(·, ·), *AW*(·, ·), and TfIdf(·, ·), respectively. In addition, *PH*_Path based on symptom-herb patterns mining is shown in Line 4–line 7, and *PDH*_Path based on symptom-herb patterns mining is shown in Line 8–Line 11.

## 5. Experiments

In this section, we conduct many experiments to evaluate the effectiveness of the proposed algorithm. We show that our proposed three step prediction approach can mine a reasonable set for each symptom on the TCM networks.

### 5.1. Experiment Setup

We first convert these datasets into heterogeneous tripartite information networks. We construct four TCM networks from TCM datasets, which consist of three types of objects: symptoms, syndromes, and herbs. Links exist between symptoms and syndromes, syndromes and herbs, and herbs and symptoms.

In order to effectively mine symptoms-herbs patterns, we adopt two kinds of strategies: *PH*_Path based strategy and *PDH*_Path based strategy. For each strategy, we apply four different measurement methods to set each term of each matrix related to this *PH*_Path (or *PDH*_Path). By combining these two kinds of strategies and four measurement methods together, we get total 8 different predicted methods. In the following section, a series of experiments will be carried on in order to find which predicted method can get the best performance.

In this work, we adopt twofold cross-validation (i.e., half training and half testing) to evaluate the performance of the prediction for each TCM network. In the training stage, we first extract two kinds of paths, symptom-herb path and symptom-syndrome-herb path. In terms of these two paths, we further build five matrices (in Section 4) according to the measurement method aforementioned (*F*, JC, AM, and TfIdf). After collecting all associated features, a training model is then built to learn the best coefficients associated with different features in deciding the symptom-herb patterns by performing multiple experiments. In the test stage, we utilize the learned coefficients to predict the potential patterns between symptoms and herbs and record whether this pattern is to appear in the test dataset.

In addition, the Insomnia and Tourette dataset lacks the object of syndrome and symptom, respectively. In this case, we assume some virtual objects (representing syndromes or symptoms) which can be constructed according to the next method. Here, we take the Insomnia dataset as an example to explain how to construct the virtual objects, namely, syndromes. First, we can get the existing patterns based on the *PDH*_Path from Infertility and Diabetes datasets, such as *p*_1_-*d*_1_-*h*_1_, *p*_2_-*d*_1_-*h*_1_; meanwhile, we can obtain the existing patterns based on the *PH*_Path from Insomnia dataset, such as *p*_1_-*h*_1_, *p*_2_-*h*_1_. Second, we can further check whether the patterns based on the *PH*_Path from Insomnia dataset exist in the dataset Insomnia or Tourette. If they exist (i.e., *p*_1_-*h*_1_, *p*_2_-*h*_1_), we can assume a virtual syndrome *d* and construct the edge between *d* and *p*_1_ and the edge between *d* and *h*_1_ (or the edge between *d* and *p*_2_). Otherwise, we only assume a virtual syndrome *d* and produce the edges between *d* and other symptoms (or the edges between *d* and other herbs). Similarly, we can construct the tripartite graph based on the Tourette dataset.

### 5.2. Evaluation Metrics

Our proposed algorithm computes a ranking score for each candidate herb and returns the top-*N* highest ranked herbs as the predicted list for a target symptom. To evaluate the prediction accuracy, we focus on how many symptoms-herbs patterns previously removed in the preprocessing step reappear in the predicted results. Therefore, we apply two popular performance metrics, namely, Precision@*N* and Recall@*N* [17–20], to capture the performance of our proposed algorithm.

Precision@*N* is the ratio of recovered symptoms-herbs patterns to the *N* predicted symptoms-herbs patterns. Recall@*N* is the ratio of recovered symptoms-herbs patterns to the set of symptoms-herbs patterns deleted in preprocessing. We divide the symptoms-herbs patterns into two sets: the test set *T*_*h*_ and the Top-*N* set *R*_*h*_. Symptoms-herbs patterns that appear in both sets are members of the hit set. Precision and Recall are defined as follows:

(10) $$\begin{array}{c} Precision=\frac{Size\;\;of\;\;Hit\;\;Set}{Size\;\;of\;\;TopN\;\;Set}=\frac{\left|T_{h}\cap R_{h}\right|}{N},\\ Recall=\frac{Size\;\;of\;\;Hit\;\;Set}{Size\;\;of\;\;Test\;\;Set}=\frac{\left|T_{h}\cap R_{h}\right|}{\left|T_{h}\right|}. \end{array}$$

### 5.3. Parameter Tuning

In our experiments, we divide each dataset into two parts: training set and test set. We further split the training data to validation data to optimize the parameters *λ*_0_, *λ*_1_, *α*, *α*_0_, *α*_1_, *β*, *β*_0_, *β*_1_, *θ*, and *K*. We have varied the neighborhood size from 10 to 50 by an interval of 10 and the other nine parameters from 0 to 1 by an interval of 0.1. Using the validation data (in Infertility dataset), we have found the best *λ*_0_ to be 0.8, *λ*_1_ to be 0.2, *α* to be 0.7, *α*_0_ to be 0.8, *α*_1_ to be 0.2, *β* to be 0.3, *β*_0_ to be 0.8, *β*_1_ to be 0.2, *θ* to be 0.5, and *K* to be 30. In addition, we have different values for these parameters in the other three datasets, but we get the similar experimental results. Here, we do not list all the values for these parameters because of the limitation of space.

**Figure 5:**
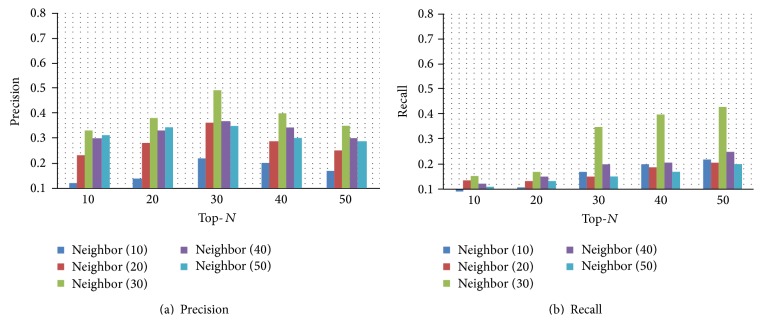
Selecting the optimal neighborhood size in Infertility Dataset. Here, in (a), the *x*-axis represents Top-*N* prediction. The *y*-axis refers to Precision. In (b), the *x*-axis represents Top-*N* prediction. The *y*-axis refers to Recall. These two figures can be obtained by using *PH*_Path based strategy, which applies the measurement method AM. In this experiment, we set *λ*_0_, *λ*_1_, and *θ* as 0.8, 0.2, and 0.5, respectively.

In Figure 5, we take the neighborhood size *K* as an example to explain how to install optimal value for each parameter. From Figure 5(a), we can see that for each Top-*N* list the Precision changes over the neighborhood size *K*. We can further observe that when the neighborhood size *K* equals 30, our proposed method gets the best performance. Also, from Figure 5(b), we have the similar results. Therefore, we set the neighborhood size *K* as 30.

### 5.4. Result and Analysis

In this section, we first evaluate the performance of four different measurement methods for two kinds of paths. And then, we compare the performance of *PH*_Path based strategy and *PDH*_Path based strategy by using the optimal measurement method.

#### 5.4.1. The Optimal Measurement Method

**Figure 6:**
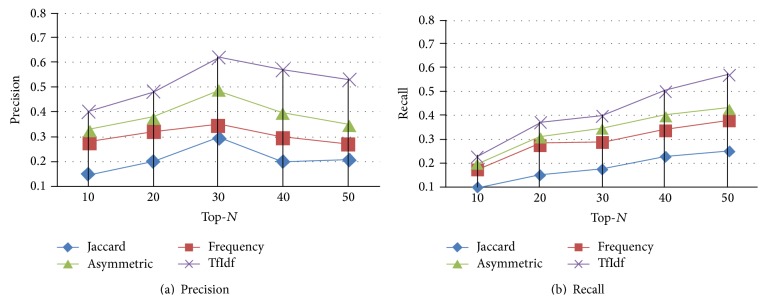
Selecting the optimal measurement method in Infertility Dataset. Here, in (a), the *x*-axis represents Top-*N* prediction. The *y*-axis refers to Precision. In (b), the *x*-axis represents Top-*N* prediction. The *y*-axis refers to Recall. These two figures can be obtained by using *PH*_Path based strategy.

**Figure 7:**
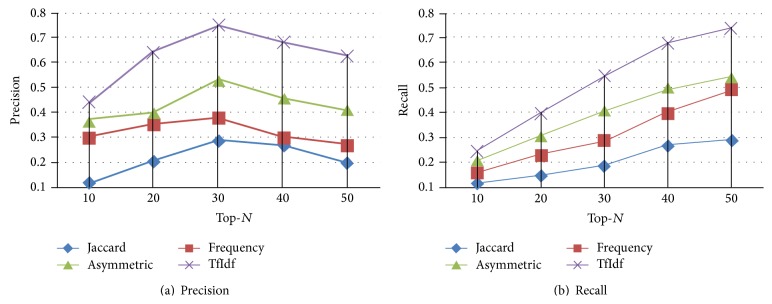
Selecting the optimal measurement method in Infertility Dataset. Here, in (a), the *x*-axis represents Top-*N* prediction. The *y*-axis refers to Precision. In (b), the *x*-axis represents Top-*N* prediction. The *y*-axis refers to Recall. These two figures can be obtained by using *PDH*_Path based strategy.

It is worth noting that a comprehensive set of experiments was conducted using every measurement method in conjunction with every evaluation metric on every dataset, and the results are very consistent across all experiments. Because of the space limitations, we show the results based on the Infertility dataset in the Figures 6 and 7. From Figure 6(a), we can see that the measurement method TfIdf apparently beats all the other three measures and produces the best prediction performance in terms of Precision. Specifically speaking, TfIdf has its average Precision  13%, 21.6%, and 30.8% better than AM, *F*, and JC, respectively. From Figure 6(b), according to Recall, TfIdf also significantly outperforms other three measures. TfIdf, respectively, achieves a 38%, a 61%, and a 116% improvement over AM, *F*, and JC. Here, an interesting result is observed that JC gets the worst performance. Contrary to JC being known to be more useful to measure the similarity between two same type of objects, it may be due to the existence of different type of objects. Similarly, from Figure 7, we can also observe that TfIdf is the best measurement method. Therefore, we should use TfIdf to help choose the best value for each term in each matrix so that the mining of symptoms-herbs patterns can produce the best results.

#### 5.4.2. The Performance of Proposed Method

In this section, we will estimate the performance of our presented Tri-TSPA based on two kinds of paths.

**Figure 8:**
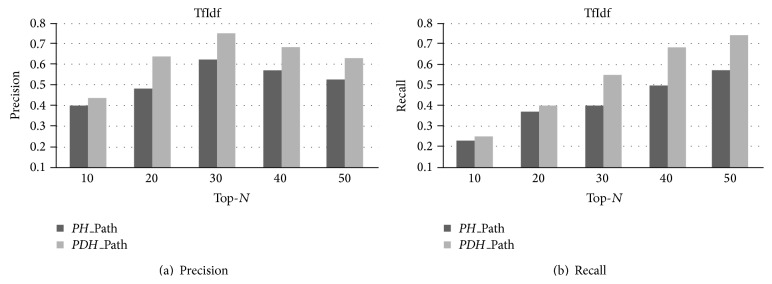
Prediction performance of our proposed method Tri-TSPA. Here, in (a), the *x*-axis represents Top-*N* prediction. The *y*-axis refers to Precision. In (b), the *x*-axis represents Top-*N* prediction. The *y*-axis refers to recall. Tri-TSPA adopts TfIdf to install the reasonable value to each term for each matrix.

First, we illustrate how our Tri-TSPA can serve as a powerful model for predicting potential symptom-herb relationships. The prediction processing performance results can be found in Figures 8(a) and 8(b). We use two prediction processing measures to evaluate the performance of each method on four TCM datasets, which are Precision at top 30 prediction results and Recall at top 30 prediction results, denoted as Precision@30 and Recall@30, respectively. In terms of these two measurements, one can observe that our proposed Tri-TSPA based on *PDH*_Path can find more symptom-herb relations than the one based on *PH*_Path, in general.

From Figure 8(a), we notice that our proposed method Tri-TSPA based on *PDH*_Path improves Precision@30 by 10.8% compared with the one based on *PH*_Path. In addition, from Figure 8(b), we also see that our proposed method Tri-TSPA based on *PDH*_Path improves Recall@30 by 11% when compared with *PH*_Path. Therefore, we can conclude that *PDH*_Path based prediction method gives a good performance overall. Here, we can see that when *N* reaches 30, the precision of both algorithms is optimal. Meanwhile, although Recall@50 of both algorithms reaches optimal value, the gap between Recall@30 of both algorithms and Recall@50 of both algorithms is very small. So we take *N* = 30 as an optimal value to achieve optimal prediction power for the Infertility dataset.

In addition to the Infertility dataset, we tested the proposed algorithm with other three datasets, and the same pattern is observed in the vast majority of cases.

#### 5.4.3. Discussion

The symptoms in TCM are related to the body as a whole. A certain subset of symptoms belongs to a certain syndrome, and the typical treatment of a syndrome usually follows a therapeutic principle, which refers to the use of a certain combination of herbs [21].

So far, we have mined a Top-*N* list of herbs for each symptom (see Table 2). However, our aim is to discover an effective combination of interacting herbs for each symptom, which is useful for healing the sick. In this section, we will introduce a matching function (MF) in order to achieve our aim.

Our matching function is as follows: first, we find all the patterns of good outcome in the dataset and then, we match the Top-*N* list with each existed pattern, and find a longest chain, namely, a maximum effective set of interacting herbs. Our matching function is described in Algorithm 2. Here, the differences between the relevant function and the matching function are as follows: the relevant function is used for filtering the bad patterns (i.e., symptom-herb); the matching function is used for finding a maximum effective set of interacting herbs for each symptom. By using MF, we get an effective combination of interacting herbs for each symptom (see Table 3). Stomachache is a manifestation of various syndromes according to Chinese medicine diagnosis. The aim of Chinese medicine is to address the root cause of disease that is a syndrome rather than a single symptom; as a result, multiple herbs are used to treat a particular syndrome. According to the assessment from a TCM practitioner, the herbs in Table 3 are appropriate to stomachache and they have the properties of relieving pain or stomach-related problems. Each of these herbs has different functions, including Regulate Qi (Nutgrass Galingale Rhizome, Tangerine Peel, Dioscoreae, Rhizoma Atractylodis Macrocephalae, Bupleurum), Regulate fluid (Plantain Seed, Tuckahoe), Clear heat (Radix Paeoniae Rubra, Chiretta), Regulate blood (Motherwort Fruit, Salvia), and Nourish Yin (Himalayan Teasel Root). Here, we think our approach works in view of TCM, because when we check the original Infertility dataset, we find that most of the combinations of our Top-*N* list of herbs exist in the original dataset.

## 6. Related Work

TCM network and its properties are researched in many fields. One of these fields is how to explore the complex relationships amongst different components of TCM clinical prescriptions. So far, there are some attempts that explicitly address this aspect.

In [22], authors proposed a new methodology of clinical decision of pulmonary tuberculosis, which can adapt the features of TCM and can be applied to other contagious diseases. This method increased the possibility and accuracy of online diagnosis and treatment especially on contagious diseases. In [23], they presented a new approach to systematically generate combinations of interacting herbs that might lead to good outcome. Their approach was tested on a dataset of prescriptions for diabetic patients to verify the effectiveness of detected combinations of herbs. Their approach is able to detect effective higher orders of herb-herb interactions with statistical validation. In this work, we also consider the factor of good outcome, but we focus on how to improve the algorithm accuracy using good outcome. In [24], they introduced a framework to explore the complex relationships amongst herbs in TCM clinical prescriptions using Boolean logic. In [25], authors put forward a framework which can be used to extract synergistic herbal combinations in a variety of clinical situations. They found that not only the herbs (present herbs) necessary for a positive outcome, but the choice of some other herbs (absent herbs) may have a negative impact on the outcome. In [5], they introduced a two-stage analytical approach. This method first uses hierarchical core subnetwork analysis to preselect the subset of herbs that have high probability in participating in herb-herb interactions and, then, detects strong attribute interactions in the preselected subset by applying MDR. In [26], a new parameter-free algorithm was designed to systematically generate a set of combinations of interacting herbs that leads to good outcome. So far, most of these researches were related to how to extract core herbs or mine herb-herb relationships, which focused on the homogeneous information networks consisting of only one type of objects. In this work, we try to extract the symptom-herb relationships based on the heterogeneous information network.

Another line similar to our research problem is the relationship mining task in heterogeneous information network [27, 28], which involves different types of objects and relations. However, these studies have a different focus compared with our work. In [27], they constructed a heterogeneous biological information network by combining multiple different databases and interaction information in order to find multidrug prescriptions that are effective and safe. In [28], they proposed MedRank, a new network-based algorithm that ranks heterogeneous objects in a medical information network. In this work, we aim at mining symptom-herb patterns in the TCM heterogeneous information network.

## 7. Conclusion

In this work, we put forward a novel three-step prediction approach to mine symptom-herb relationships effectively and efficiently. Experiments on the TCM network show that our method can find symptom-herb relationships with much higher accuracy using heterogeneous topological features. The results have shown that the performance is indeed superior when the symptoms are mapped to herbs via syndromes, rather than a direct mapping between symptoms and herbs. In other words, syndrome differentiation (patient classification) is a crucial step to a successful treatment in TCM. In the future, we intend to extend our work in the following three directions. Firstly, a new measure to estimate the performance in the proposed method should be explored. Secondly, another novel similarity measure method should be studied to capture the rich topological features. Thirdly, a new matching function to improve the predictive performance should be sought.

## Figures and Tables

**Algorithm 1:**
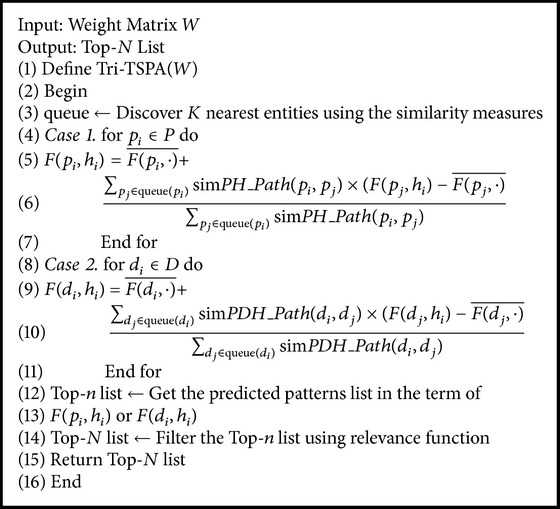
Tri-TSPA.

**Algorithm 2:**
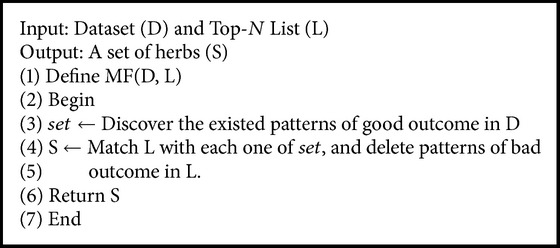
MF.

**Table 1:** Properties of four TCM data sets. Here, “—” represents that this attribute can not be included in this data set.

	Insomnia	Infertility	Diabetes	Tourette
Number of prescriptions	460	852	1674	670
Number of herbs	111	251	204	189
Number of symptoms	155	389	186	—
Number of syndromes	—	106	178	98
Symptoms per herb	82.58	71.64	84.72	—
Syndromes per herb	—	24.34	29.56	20.56
Herbs per symptom	59.14	46.4	33.89	—
Herbs per syndrome	—	57.41	92.91	71.13

**Table 2:** An Example of Top-30 List. This table can be obtained by using *PDH*_Path based strategy. Here, the third column represents symptom-herb ranking rating produced by Algorithm 1.

Symptom	Herb	Rating
Stomachache	Chiretta	7.567
	Radix Paeoniae Rubra	6.765
	Bupleurum	6.70
	Ligustrum Japonium	6.43
	Epimedium Herb	6.397
	Paeonia sterniana Fletcher in Journ	6.396
	Radix Polygoni Multiflori	6.167
	Rhizoma Atractylodis Macrocephalae	6.0
	Salvia	5.989
	Astragali Radix	5.973
	Tuckahoe	5.915
	Licorice Roots Northwest Origin	5.899
	Dioscoreae	5.659
	Homo sapiens	5.549
	Rehmannia root	5.438
	Motherwort Fruit	5.357
	Tortoise Shell	5.347
	Himalayan Teasel Root	5.327
	Tangerine Peel	5.209
	Nutgrass Galingale Rhizome	5.176
	Palmleaf Raspberry Fruit	5.165
	Diverse Wormwood Herb	4.97
	Plantain Seed	4.934
	Bitter Orange	4.92
	Safflower	4.905
	Hyacinth Bean	4.876
	Finger Citron	4.844
	Towel Gourd Vegetable Sponge	4.819
	Common Macrocarpium Fruit	4.736
	Zedoary	4.736

**Table 3:** An effective combination of interacting herbs for symptom *Stomachache*. Based on Table 2, this table can be obtained by using Algorithm 2.

Symptom	Herb	Rating
Stomachache	Chiretta	7.567
	Radix Paeoniae Rubra	6.765
	Bupleurum	6.70
	Ligustrum Japonium	6.43
	Epimedium Herb	6.397
	Paeonia sterniana Fletcher in Journ	6.396
	Rhizoma Atractylodis Macrocephalae	6.0
	Salvia	5.989
	Tuckahoe	5.915
	Licorice Roots Northwest Origin	5.899
	Dioscoreae	5.659
	Motherwort Fruit	5.357
	Himalayan Teasel Root	5.327
	Tangerine Peel	5.209
	Nutgrass Galingale Rhizome	5.176
	Palmleaf Raspberry Fruit	5.165
	Plantain Seed	4.934
	Hyacinth Bean	4.876
	Common Macrocarpium Fruit	4.736

## Notations

*P*: Symptom
*D*: Syndrome
*H*: Herb
*PH*_Path: The path of symptom-herb
*PDH*_Path: The path of symptom-syndrome-herb
Sim*PH*_Path: The similarity based on *PH*_Path
Sim*PP*: The similarity based on *P*-*P* matrix
Sim*PH*: The similarity based on *P*-*H* matrix
Sim*PDH*_Path: The similarity based on *PDH*_Path
Sim*PD*: The similarity based on *P*-*D* matrix
Sim*DH*: The similarity based on *D*-*H* matrix
Sim*DD*: The similarity based on *D*-*D* matrix.
